# Intratympanic injection of dexamethasone for management of labyrinthitis associated with COVID-19 disease resistant cases

**DOI:** 10.1016/j.amsu.2022.104429

**Published:** 2022-08-17

**Authors:** Wael F. Ismaiel, Mohamed H. Abdelazim, Ashraf A. Wahba, Mahmoud E. Alsobky, Ahmed Abdalrhman Ibrahim, Soliman A. Ghanem, Ali A. Rabaan, Elshahat I. Ismail

**Affiliations:** a Otorhinolaryngology Department, Faculty of Medicine, Al-Azhar University, Damietta, Egypt; b Audiology Unit, Otorhinolaryngology Department, Faculty of Medicine, Al-Azhar University, Damietta, Egypt; c Molecular Diagnostic Laboratory, Johns Hopkins Aramco Healthcare, Dhahran, Saudi Arabia; d College of Medicine, Alfaisal University, Riyadh, Saudi Arabia; e Department of Public Health and Nutrition, The University of Haripur, Haripur, Pakistan; f Audiology Unit, Otorhinolaryngology Department, Faculty of Medicine, Mansoura University, Mansoura, Egypt

**Keywords:** COVID-19, Dexamethasone, Hearing loss, Labyrinthitis

## Abstract

**Objective:**

To evaluate the efficacy of mixed oral prednisolone and intratympanic dexamethasone (ITID) injection in labyrinthitis, due to COVID 19.

**Methods:**

Seventy-five post-COVID-19 labyrinthitis patients were included. Those patients were treated with systemic oral prednisolone for two weeks and ITID. Patients who refuse ITID were ordered to continue oral prednisolone treatment. Assessment of outcome and audiometry for hearing evaluation was done 1, 2 and 4 weeks as well as 3 months post-treatment.

**Results:**

Patients in oral steroid only group were 26 patients, while patients in oral steroid and ITID group were 49 patients. In oral steroid group; 11/26 patients showed complete recovery, 3/26 had partial recovery and 12/26 not recovered. In other group; 38/46 patients had complete recovery, 6 had partial recovery and 5/49 patients not recovered.

**Conclusion:**

Combined systemic corticosteroid with ITID showed a marked improvement of post-COVID vestibular disorder and hearing loss than only using oral corticosteroid therapy.

## Highlights

• Audiovestibular symptoms in COVID-19 patients include labyrinthitis hearing loss cording to reports.
• 75 post-COVID-19 labyrinthitis patients were treated with systemic oral prednisolone for two weeks ± Intratympanic injection of corticosteroid.
• Patients who refuse Intratympanic injection were ordered to continue oral prednisolone treatment.
• It showed marked improvement of post- COVID labyrinthitis and hearing loss symptoms more than oral corticosteroid therapy.

## 1 Introduction

Hearing loss and vestibular dysfunction are commonly caused by viral infections. These viruses include *Herpesviridae members,* such as varicella-zoster, herpes simplex, Epstein-Barr, and cytomegalovirus. Also, *Paramyxoviridae,* such as measles, mumps, and parainfluenza viruses. In addition, hepatitis, influenza, polio, rubella, and human immunodeficiency viruses are involved [1]. The following mechanisms are suspected of causing viral-induced sensorineural hearing loss (SNHL), dizziness, vertigo, imbalance, or tinnitus; Direct invasion and damage to the inner ear structures, including the vestibulocochlear nerve and the organ of corti [2]; Injury and inflammation caused by the immune system, including neuroinflammation [3,4]; reactivation of a latent viral infection inside the inner ear [5]. Also, viruses can cause infection of the middle ear, resulting in hearing loss due to middle-ear effusion. The role of coronaviruses has not been systemically studied yet, although they commonly cause middle-ear infection [6]. COVID-19 is a respiratory and vascular disease that is highly infectious and causes anosmia [7] and ageusia [8], highlighting its sensory systems tropism. Although some recent reports of audiovestibular symptoms in COVID-19 patients rely on self-reported hearing loss, they did not comment on hearing prognosis following COVID-19 resolution. These reports lacked the objective documentation of SARS-COV-2 testing on the included patients [9]. There is a controversy over the influencing pathogenesis of COVID-19 leading to a cautious use of dexamethasone and prednisolone in the treatment of COVID-19 infection; however this severe caution does not excist when using cortisone in post COVID-19 [10,11]. For SNHL patients, intratympanic low dose steroids are increasingly used [12]. To overcome this lack, the current study aims to evaluate our experience in the intratympanic injection of dexamethasone in audiovestibular labyrinthitis due to COVID 19 cases after systemic treatment failures.

## 2 Patients and methods

The current research comprised audiovestibular labyrinthitis patients after recovery from COVID-19 infection from December 2020 to March 2022. It was conducted at the Otorhinolaryngology Department, Al-Azhar Faculty of Medicine, Damietta, and the Otorhinolaryngology Department, the audiology unit, Faculty of Medicine, Mansoura University, Egypt.

The Institutional Review Board (IRB-00012367-20-07-008) at Al-Azhar faculty of medicine, Damietta, approved this study and registered in registry website and the manuscript was written following the STROCSS checklist [13].

Patients with sensorineural hearing loss 30 dBHL or more in three consecutive frequencies within 72 h and rotatory vertigo for days, diagnosed as post-COVID-19 labyrinthitis and aged 18 years or older, were included in this study, while patients with different causes of hearing loss and who have sensitivity to steroids were excluded. Also, patients with neurological or medical condition affecting the auditory system, otoscopic evidence of ear-drum abnormalities, hypertensive, ototoxic drug intake, diabetic, noise exposure, peptic ulcer, chronic middle ear pathology, or previous ear surgery were excluded.

### 2.1 Sample collection

Seventy-five patients of unilateral post-COVID labyrinthitis suffering from different sensorineural hearing loss degrees were included. The infection onset, duration, and any remaining complications after recovery were recorded. Those patients were treatment systemically by oral methylprednisolone and ITID called oral steroids and ITID group. Patients who refuse ITID were continue treated by oral methylprednisolone called oral steroids group.

### 2.2 Diagnosis

The COVID-19 diagnosis was confirmed by PCR. The labyrinthitis diagnosis was made following the criteria; a history of severe and continuous rotatory vertigo, postural instability, and nausea that began sub-acutely or acutely (within minutes to hours). In addition, SNHL (250–8000 Hz) and normal middle ear function were reported on clinical examination. Caloric irrigation showed a hypo-responsiveness or absent response in the affected horizontal ear canal. Additionally, toward the unaffected ear (fast phase), there was spontaneous horizontal nystagmus with a rotating component but no indication of a central vestibular affection. An ipsilateral deficit of the horizontal semicircular canal was revealed by the head-thrust test (the patient's head turn quickly to the right and left to stimulate compensatory eye movements). The asymmetry between the two sides should be more than 20%, according to Jongkees's formula for vestibular paresis [14].

Equipment:

1. Two-channel audiometer, Interacoustic AC40 diagnostic audiometer, version 1.48 (Denmark).
2. Immittancemetry, interacoustic, AT 235 (Denmark).
3. Video nystagmography, Micro-medical, Spectrum, Visual eye, version 6.1. (USA).
4. Locally made sound-treated room.
5 Biologic Auditory Evoked Potential, Navigator Pro, version 7.2.1 (Natus Medical, Inc., San Carlos, CA, USA).

### 2.3 Procedure

The treatment started within the first week of affection. All participants were given antiemetic drugs, 50–150 mg dimenhydrinate daily for three days, and systemic steroids throughout the disease's acute stage. Methylprednisolone (1 mg/kg/day) tablet was given for one week. Then, a gradual withdrawal over the next week was made. Also, an H2 blocker once/day was used to save the gastric mucosa. Three injection administered over alternative days with dexamethasone (10 mg/ml) in the affected ear until no improvement was recorded by pure tone audiometry (after every three intratympanic dexamethasone injections) or complete improvement occurred; this group termed steroids and ITID group. The patients refused intratympanic dexamethasone injection, continued on systemic treatment, and were used for comparison with the IT injection group and called steroids group.

The intratympanic injection of dexamethasone was done in outpatient clinic and under local anesthesia by xylocaine spray of tympanic membrane, the patient on supine position with head tilted to opposite side, the dexamethasone loaded to 1 ml syringe connected to 25 gouge needle then we inject from 0.3 to 0.7 ml of dexamethasone to middle ear through posterosuperior part of tympanic membrane and the patient stay in position for about 30 min.

All patients who participated in this study were evaluated four times, initial evaluation at the first visit and after two weeks, one month, and three months of treatment.

Cervical vestibular evoked myogenic potentials (cVEMPs) and caloric tests were used to measure unilateral vestibular weakness. The extent of canal paresis was measured using caloric irrigation with water at 30 °C and 44 °C. The vestibular paresis formula by Jongkees was used to calculate caloric lateralization as follows:

{[(R30° + R44°) − (L30° + L44°)] ÷ (R30° + R44° + L30° + L44°)} × 100

where R indicates right and L left, and ° is °C. Our vestibular laboratory norms recorded abnormal caloric findings for 20% or higher caloric lateralization.

Regarding the treatment outcome, patients were categorized into three groups; complete to good response (≥30 dBHL improvement or ≥ 30% improvement in the word recognition score); partial or moderate response (≥10 to < 30 dBHL improvement or ≥ 10% to < 30% improvements in the word recognition score); poor or no-response (≤9 dBHL improvements or ≤ 9% improvement in the word recognition score) [15].

Complete vestibular disorder resolution was considered if the dizziness handicap inventory (DHI) score was less than 6, caloric lateralization was less than 20%, and the VEMPs were normal.

### 2.4 Statistical analysis

Statistical analyses were performed using SPSS v23 statistical software (SPSS, Inc, Chicago, Illinois). Descriptive statistics (means correlation standard deviations) were calculated for quantitative variables. Two-sided Chi-square, student-t and ANOVA test were used as appropriate for parametric data, and Mann-Whitney U and Kruskal Wallis tests were employed for non-parametric variables. The significance level was calculated and P ≤ 0.05 was considered statistically significant, while P > 0.05 was considered statistically non-significant.

## 3 Results

This research comprised 75 patients with unilateral post-COVID labyrinthitis 34 males (45.3%) and 41 females (54.7%), and their ages ranged between 21 and 69 years. Patients in oral steroid group continue oral steroid treatment were 26 patients (34.7%), while patients in oral steroid and ITID group were 49 (65.3%) patients. In oral steroid group; 11/26 patients (42.3%) showed complete recovery, 3/26 (11.5%) had partial recovery and 12/26 (46.2) had no recovery. In oral steroid and ITID group; 38/46 patients (77.6%) had complete recovery, 6 (12.2%) had partial recovery and only 5/49 patients (10.2%) had no recovery (see Table 1).

Hearing loss severity was classified as mild, moderate, severe and profound and it showed a statistically significant difference between the studied groups (P < 0.001) (Table 2).

**Table 1:** Patients' characteristic and treatment outcome of the COVID-19 patients.

Group	Total		Oral steroid group		Oral steroid and ITID group	
			Mean ± SD	Range	Mean ± SD	Range
Age (years)			49.5 ± 12.7	21–69	51.8 ± 10.8	35–69
Gender	No	%	No	%	No	%
Males	34	45.3	11	42.3	23	46.9
Females	41	54.7	15	57.7	26	53.1
Total	75	100	26	34.7	49	65.3
Risk factors^b^						
Diabetes	50	66.7	18	69.2	32	65.3
Chronic sinusitis	32	42.7	13	50.0	19	38.8
Recurrent ear infection	23	30.7	8	30.8	15	30.6
Improvement			No	%	No	%
Complete	49	65.3	11	42.3	38	77.6
Partial	9	12.0	3	11.5	6	12.2
Not improved	17	22.7	12	46.2	5	10.2
Statistical test			χ^2^	P value	χ^2^	P value
Significance^a^			0.037	0.647	1.878	0.001*

*p = 0.001: highly significant.

a χ^2^: Chi square in comparison between improved and non-improved cases, ITID: intratympanic injection of dexamethasone.

b Patients may have more than one risk factor.

**Table 2:** Degree of hearing loss and treatment outcome of the COVID-19 patients.

Hearing loss classification		Oral steroid group (n = 26)				Oral steroid and ITID group (n = 49)				Significance of success	
		Success		Failed		Success		Failed			
Degree	N	No	%	No	%	No	%	No	%	χ^2^	P
Mild	10	8	30.8	1	3.75	1	2.04	0	0.00	−18.81	0.000*
Moderate	36	5	19.2	6	23.1	24	49.0	1	2.04	−0.887	0.007*
Severe	29	1	3.85	5	19.2	19	38.8	4	8.16	21.24	0.000*
Profound	75	14	53.8	12	46.2	44	89.8	5	10.2	0.867	0.007*

*p < 0.001: highly significant in comparison of success rate, χ^2^: Chi square, ITI: intratympanic injection.

Regarding the final recovery time after systemic steroid treatment (oral steroid group) had recovery in 14/26 patients (53.8%); 4/14 cases (28.6%) in the first week, 5/14 cases (35.7%) in two weeks, and 5/14 cases (35.7%) in the fourth week, while oral steroid and ITID group had recovery in 44/49 patients (89.8%). Of them, 18/44 cases (40.9%) had recovered in the first week, 15/44 patients (34.1%) had removed in the 2nd week, 9/44 cases (20.5%) had recovered within 4 weeks and only 2/44 cases had recovered 3 months (Table 3).

**Table 3:** Time of recovery from labyrinthitis after treatment of the COVID-19 patients.

Recovered cases at follow-up	Oral steroid group (N = 14)		Oral steroid and ITID group (N = 44)		Test of Significance	
	No	%	No	%	χ^2^	P
First week	4	28.6	18	40.9	0.634	0.009*
Two weeks	5	35.7	15	34.1	−0.015	0.913
Four weeks	5	35.7	9	20.5	−0.523	0.016*
Three months	0	0.00	2	4.55	0.331	0.038*

*p < 0.001: highly significant, χ^2^: Chi square, t: paired *t*-test, ITI: intratympanic injection, N/A: not applicable.

As regard vestibular disorders; complete resolution was observed in 13 (50%) of cases in oral steroid group and 44 (89.8%) of cases in oral steroid and ITID group. Comparison between them showed statistically significant difference (p = 0.008) as shown in table (4).

**Fig. 1:**
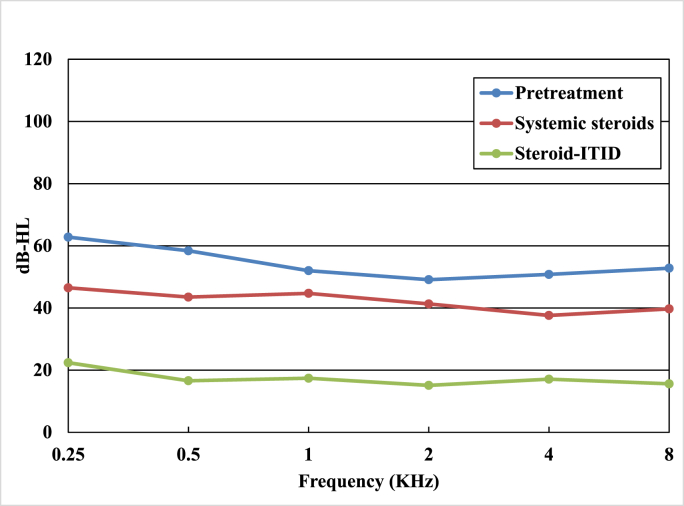
Pre-treatment and post-treatment audiometric criteria of the two groups.

Oral steroid group showed significantly higher complications (21.5 ± 8.83) compared with oral steroid and ITID group which as (2.63 ± 1.41) with statistically highly significant difference (P < 0.001) in comparison between the two groups (Table 4). Also, there was a marked improvement of pure tone audiometry in oral steroid and ITID group than oral steroids group (P < 0.001) (Table 5 and Fig. 1) (see Table 6).

**Table 4:** Comparison between the two groups as regard treatment of vestibular disorders.

Vestibular disorders	Total		oral steroid group (N = 26)		Oral steroid and ITID group (N = 49)		Test of Significance	
	No	%	No	%	No	%	χ^2^	P
Complete resolution	57	79.0	13	50.0	44	89.8	0.737	0.008*
Incomplete resolution	18	24.0	13	50.0	5	10.2	−0.958	0.001*
Total	75	100	26	100	49	100		

*p < 0.001: highly significant, χ^2^: Chi square, t: paired *t*-test, ITID: intratympanic injection of dexamethasone.

**Table 5:** Treatment complications before and after treatment of labyrinthitis.

Treatment complication	Oral steroid group				Oral steroid and ITID group				Significance after treatment	
	Before (50)		After		Before (29)		After			
	No	%	No	%	No	%	No	%	χ^2^	P
Pain	36	72.0	25	50.0	25	86.2	3	10.3	28.34	0.000*
Ear fullness	28	56.0	22	44.0	22	75.9	2	6.9	21.52	0.000*
Vertigo	23	46.0	15	30.0	15	51.7	1	3.4	18.73	0.000*
Headache	50	100	38	76.0	29	100	5	17.2	39.12	0.000*
Dizziness	26	52.0	12	24.0	12	41.4	1	3.4	13.28	0.000*
Tinnitus	28	56.0	15	30.0	15	51.7	4	13.8	10.37	0.000*
Infection	20	40.0	16	32.0	16	55.2	2	6.9	17.95	0.000*
Hearing loss	50	100	29	48.0	29	100	3	10.3	22.41	0.000*
Average	32.6 ± 11.7		21.5 ± 8.83		20.4 ± 6.76		2.63 ± 1.41		t: 16.8	0.000*

*p < 0.001: highly significant, χ^2^: Chi square, t: paired *t*-test, ITI: intratympanic injection.

**Table 6:** Comparison of pretreatment and post-treatment pure tone audiometry of the affected ear of the studied patients.

PTA (KHz)	Pre-steroid treatment	Post-steroid treatment		Significance	
		Oral steroid group	Oral steroid and ITID Group	*t*-test^a^	P value
0.25	62.8 ± 9.99	46.5 ± 11.44	22.4 ± 8.172	7.621	0.000*
0.50	58.4 ± 11.95	43.5 ± 11.00	16.6 ± 9.499	7.925	0.000*
1.0	52.0 ± 11.47	44.7 ± 12.09	17.4 ± 9.368	8.235	0.000*
2.0	49.1 ± 11.81	41.3 ± 10.48	15.1 ± 9.258	6.452	0.001*
4.0	50.8 ± 14.96	37.6 ± 12.54	17.1 ± 9.694	6.517	0.001*
8.0	52.8 ± 14.39	39.7 ± 16.36	15.6 ± 16.17	5.644	0.001*

*P < 0.001 = statistically highly significant.

a Comparison between systemic steroid treatment and intratympanic injection of dexamethasone (ITID).

## 4 Discussion

Currently, the COVID-19 virus effect on the sinuses, ear and hearing organs needs more investigation [16,17]. There are multiple treatments for labyrinthitis, starting from systemic steroids, intratympanic injection of steroids, and antibiotics, until cochlear implantation, an effective treatment for sensorineural hearing loss [18]. The effectiveness of the corticosteroid for treating labyrinthitis and sudden SNHL is lacking [19]. It is more efficient to use intratympanic steroids, either alone or in combination with systemic steroids, than systemic steroids to treat sudden hearing loss [20]. Because of suspected viral infections, antiviral drugs may be useful in treating labyrinthitis, although there is no better outcome evidence when antivirals are combined with systemic steroids [21].

We had 75 patients with post-COVID 19 labyrinthitis, and females were more susceptible than males. Taxak et al. [18] agreed on this; females are 1.5 times more vulnerable to infection than males.

No particular COVID-19 therapy was recommended if there were no respiratory symptoms. To treat the labyrinthitis, oral corticosteroids for one-week daily was initiated, supported by physiotherapy, and followed by a progressive clinical improvement. Corticosteroids were efficient in acute vestibular vertigo treatment [17]. Nevertheless, data on the cortico-therapy efficacy in COVID-19 related otitis are limited. Oral corticosteroids alone or in combination with intratympanic dexamethasone injections had been proposed with an increasing clinical improvement in most cases [19,20].

There is a marked hearing improvement after combination of systemic steroids and ITID than oral corticosteroids only in the current study, with a highly significant difference (P < 0.001).

In agreement of our results, Koumpa et al. [21] and Chern et al. [22] found that intratympanic and oral steroids mix improved hearing threshold and word recognition scores. Additionally, Turkish research reported a recovery of normal hearing following the oral hydroxychloroquine administration [23].

Corticosteroid usage showed better results in the sudden sensorineural hearing loss (SSNHL) treatment [24]. Despite the controversy on the steroid usage for COVID-19 patients, intratympanic steroids could be utilized to treat COVID-related SSNHL [25]. Rahman and Wahid [26] reported hearing improvement after three times intratympanic steroid injections in five days in an asymptomatic patient tested for COVID-19 by RT-PCR. In contrast, Lang et al. [27] observed no significant improvement with oral steroid therapy.

Also, it is reported by Rahman and Wahid [26] that an asymptomatic patient went to their clinic with sensorineural hearing loss and a positive swab for COVID-19. This patient was treated with intratympanic steroids that resulted in a modest improvement. He was previously healthy and had no other identifiable reasons for his SSNHL. The benefit was from the oral steroids with no further benefit from the intratympanic steroid injections [21]. However, other studies reported unilateral sudden moderate to severe high-frequency SNHL and tinnitus, with no or partial improvement after intratympanic steroid [[28], [29]].

On the other hand, Lai et al. [30][ concluded that intratympanic and systemic steroids’ therapies appear to show similar short-term efficacy for restoring hearing in patients with idiopathic SSNHL. Intratympanic therapy may reduce systemic side effects associated with steroid use. However, another recent study by de Cates C & Winters [31]stated that SSNHL has high rates of spontaneous recovery in ITID; reported rates range from 32 to 65%. ITID treatment modality needs to be used cautiously due to a lack of confirmed understanding of the underlying etiology of some inner ear diseases.

The marked improvement of vestibular disorders in the oral steroid and ITID group may be attributed to the recovery of vestibular hair cells, which may be affected more than the vestibular nerve fiber in post COVID labyrinthitis. We used combination IT injection and oral steroids in post COVID labyrinthitis due to IT injection improve cochlear and vestibular hair cells, cochlear structure and vestibular end organ while oral steroid act mainly on vestibular nerve fiber.

Limitations include the small sample size of oral steroid group.

## 5 Conclusion

Combined systemic corticosteroid with intratympanic injection of corticosteroid showed a marked improvement of post-COVID vestibular disorders and hearing loss than only using oral corticosteroid therapy. However, more future studies are recommended to confirm these results and clear the controversy about using ITID especially in COVID-19 labyrinthitis.

## Ethical approval

Ethical approval was obtained from Azhar Damietta Faculty of Medicine, number: IRB-00012367-20-07-008.

## Sources funding

This study did not receive any funding from governmental or private organizations.

## Author contributions

Study concept or design: WFI, MHA, AAW, MEA, SAG, EII.

Data collection: WFI, MHA, AAW, SAG, EII, AAR.

Data interpretation: WFI, MHA, AAW, MEA, AAI, SAG, AAR.

Literature review: WFI, MHA, AAW, MEA, AAI, SAG, EII, AAR.

Data analysis: WFI, MHA.

Drafting of the paper: ALL.

Editing of the paper: ALL.

Manuscript revision: ALL.

## Registration of research studies

IClinicalTrials.gov Identifier:

## Guarantor

Dr. Wael Ismaiel.

## Consent

Electronic written informed consent was obtained from participants for publication of this study.

## Provenance and peer review

Not commissioned, externally peer-reviewed.

## Conflicts of interest

The authors declare no conflict of interest.
